# Sirt1–hypoxia‐inducible factor‐1α interaction is a key mediator of tubulointerstitial damage in the aged kidney

**DOI:** 10.1111/acel.12904

**Published:** 2019-01-06

**Authors:** Dong Ryeol Ryu, Mi Ra Yu, Kyoung Hye Kong, Hyoungnae Kim, Soon Hyo Kwon, Jin Seok Jeon, Dong Cheol Han, Hyunjin Noh

**Affiliations:** ^1^ School of Medicine Ewha Womans University Seoul Korea; ^2^ Hyonam Kidney Laboratory Soon Chun Hyang University Seoul Korea; ^3^ Department of Internal Medicine Soon Chun Hyang University Seoul Korea

**Keywords:** age, deacetylation, HIF‐1α, Sirt1

## Abstract

Although it is known that the expression and activity of sirtuin 1 (Sirt1) decrease in the aged kidney, the role of interaction between Sirt1 and hypoxia‐inducible factor (HIF)‐1α is largely unknown. In this study, we investigated whether HIF‐1α could be a deacetylation target of Sirt1 and the effect of their interaction on age‐associated renal injury. Five‐week‐old (young) and 24‐month‐old (old) C57Bl/6J mice were assessed for their age‐associated changes. Kidneys from aged mice showed increased infiltration of CD68‐positive macrophages, higher expression of extracellular matrix (ECM) proteins, and more apoptosis than young controls. They also showed decreased Sirt1 expression along with increased acetylated HIF‐1α. The level of Bcl‐2/adenovirus E1B‐interacting protein 3, carbonic anhydrase 9, Snail, and transforming growth factor‐β1, which are regulated by HIF‐1α, was significantly higher in aged mice suggesting that HIF‐1α activity was increased. In HK‐2 cells, Sirt1 inhibitor sirtinol and siRNA‐mediated knockdown of Sirt1 enhanced apoptosis and ECM accumulation. During hypoxia, Sirt1 was down‐regulated, which allowed the acetylation and activation of HIF‐1α. Resveratrol, a Sirt1 activator, effectively prevented hypoxia‐induced production of ECM proteins, mitochondrial damage, reactive oxygen species generation, and apoptosis. The inhibition of HIF‐1α activity by Sirt1‐induced deacetylation of HIF‐1α was confirmed by Sirt1 overexpression under hypoxic conditions and by resveratrol treatment or Sirt1 overexpression in HIF‐1α‐transfected HK‐2 cells. Finally, we confirmed that chronic activation of HIF‐1α promoted apoptosis and fibrosis, using tubular cell‐specific HIF‐1α transgenic mice. Taken together, our data suggest that Sirt1‐induced deacetylation of HIF‐1α may have protective effects against tubulointerstitial damage in aged kidney.

## 1 INTRODUCTION

Aging is a global health burden worldwide. The kidney is a target organ affected by aging‐associated changes, such as glomerulosclerosis, tubular atrophy, interstitial fibrosis, and arteriosclerosis (Choudhury & Levi, 2011; Rule et al., 2010). The aged kidney shows significantly increased vulnerability to challenges and impaired ability to recover from acute kidney injury, contributing to the development of chronic kidney disease (Epstein, 1996). Hypoxia is a well‐known inducer of acute kidney injury, and it has also been shown to increase in the aged kidney, especially in the cortex (Tanaka et al., 2006). Since hypoxia‐inducible factor‐1 α(HIF‐1α) is a central regulator of cellular adaptation to hypoxia, it is plausible that chronic activation of HIF‐1α can occur in the aged kidney. Although HIF‐1α appears to exert a protective effect on renal damage in acute kidney injury (Bernhardt et al., 2006; Ma et al., 2009; Matsumoto et al., 2003; Weidemann et al., 2008), chronic sustained activation of HIF‐1α in renal tubular epithelial cells has been found to promote epithelial to mesenchymal transition and kidney fibrosis (Higgins et al., 2007).

Sirtuin 1 (Sirt1), a mammalian homolog of yeast silent information regulator 2 (Sir2), is a survival factor that is involved in lifespan extension (Dali‐Youcef et al., 2007; Imai, Armstrong, Kaeberlein, & Guarente, 2000). Previous studies have reported that Sirt1 mediates a wide range of cellular responses through its deacetylation activity targeting numerous transcription factors such as p53, forkhead box O 3 (Foxo3), nuclear factor‐κB (NF‐κB), and peroxisome proliferator‐activated receptor gamma coactivator‐1α (PGC)‐1α (Feige & Auwerx, 2008). Recently, it has been reported that Sirt1 binds to HIF‐1α and deacetylates its lysine residues (Joo et al., 2015; Laemmle et al., 2012; Lim et al., 2010). However, the effect of Sirt1–HIF‐1α interaction on the activity of HIF‐1α remains unclear and whether the interplay between Sirt1 and HIF‐1α would be protective or harmful in the aged kidney has not been reported. Given that both HIF‐1α and Sirt1 play important roles in the cellular response to hypoxic stress in the aged kidney, we propose that the balance between them modulates the acetylation status and activity of HIF‐1α by which the latter mediates the progression of age‐associated renal damage.

## 2 RESULTS

### 2.1 The aged kidney showed increased extracellular matrix production, macrophage infiltration, and apoptosis

**Figure 1:**
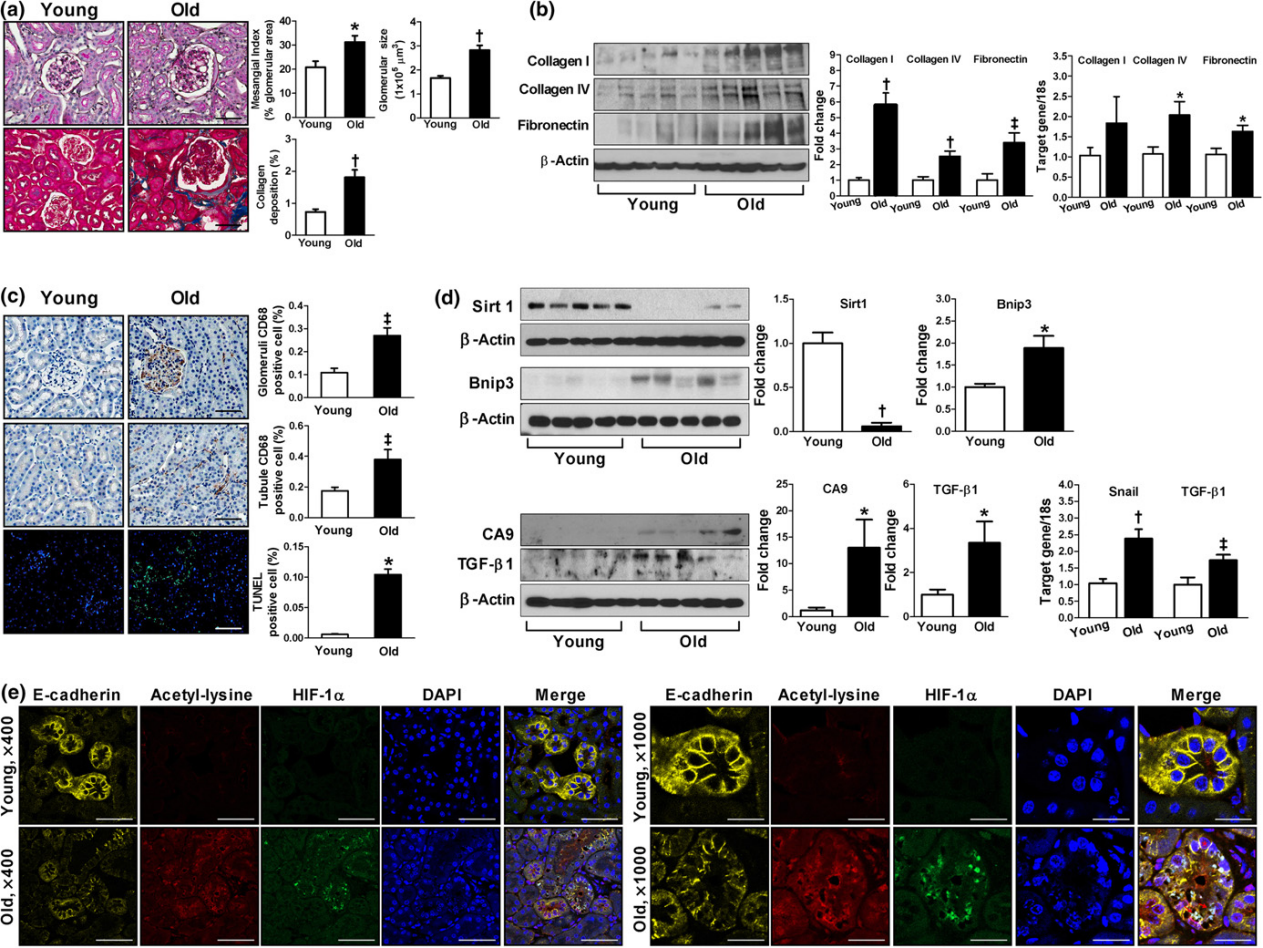
Age‐induced changes of kidney. Representative kidney sections stained with PAS (a), Masson's trichrome (a), immunohistochemical staining for CD68‐positive macrophages (c), TUNEL staining (c), and immunofluorescence staining for E‐cadherin, acetyl‐lysine, and HIF‐1α (e). Note acetylated HIF‐1α‐positive cells with a loss of E‐cadherin (arrow) compared to negative cells with intact E‐cadherin expression (asterisk) in e; bars = 50 µm (a), 50 and 100 µm (c), and 50 and 20 µm (e), respectively. (b, d) Collagen I and IV, fibronectin, Sirt1, Bcl‐2 adenovirus E1B‐interacting protein 3 (Bnip3), carbonic anhydrase 9 (CA9), and TGF‐β1 protein expression levels were analyzed using immunoblot analysis. Real‐time RT–PCR was performed to measure the level of mRNA expressions of collagen I and IV, fibronectin, Snail, and TGF‐β1. *p < 0.05, ^†^ *p* < 0.001, and ^‡^ *p* < 0.01 vs. young mice, *n* = 5–7

We first examined the functional and structural changes of the aged kidney. As shown in Table 1, aged mice showed higher kidney/body weight ratio and albuminuria as compared with young mice. Glomerular filtration rate (GFR) was significantly lower in the aged mice than in the young control mice. However, blood urea nitrogen (BUN), creatinine, and cystatin C levels were not different between the two groups. Histological analysis exhibited increases in the mesangial matrix area and glomerular size in the aged mice compared to the young mice (Figure 1a). The expression of type I/IV collagen and fibronectin was significantly higher in the renal cortex of the aged mice compared with the young controls (Figure 1b). Masson's trichrome staining confirmed a higher deposition of collagen in the aged mice than in the young mice (Figure 1a). As shown in Figure 1c, the aged mice exhibited a significantly higher number of CD68‐positive cells and terminal deoxynucleotidyl transferase dUTP nick end labeling (TUNEL)‐positive apoptotic cells than the young controls.

**Table 1:** Characteristics of young (5‐week‐old) and aged (24‐month‐old) mice

	Young	Aged
Body weight (g)	24.3 ± 0.1	38.1 ± 0.4*
Kidney weight/body weight (%)	0.77 ± 0.01	0.86 ± 0.03†
BUN (mg/dl)	39.1 ± 2.0	42.2 ± 2.5
Creatinine (mg/dl)	0.18 ± 0.01	0.93 ± 0.44
Cystatin C (mg/dl)	0.39 ± 0.01	0.48 ± 0.06
Urine volume (ml/day)	2.5 ± 0.3	2.0 ± 0.2
Urine albumin (µg/day)	1.12 ± 0.10	1.51 ± 0.16†
GFR (dl/hr)	0.08 ± 0.01	0.04 ± 0.01‡

Notes

BUN: blood urea nitrogen; GFR: glomerular filtration rate.

Data are mean ± *SE*,

^*^ *p* < 0.001, ^†^ *p* < 0.05, ^‡^ *p* < 0.005, vs. young mice.

### 2.2 **The aged kidney showed decreased Sirt1 expression but increased HIF‐1**α** expression and its acetylation**

Since both Sirt1 and HIF‐1α play critical roles in cellular adaptation to chronic hypoxic stress in the aging process, we examined the expressions of Sirt1 and HIF‐1α in the aged kidney. As shown in Figure 1d,e, the aged kidney showed significantly lower expression of Sirt1 but higher expression of HIF‐1α, mostly in the acetylated form. Of note, the expression of acetylated HIF‐1α was observed in fragmented nuclei rather than an intact one and was associated with a loss of E‐cadherin, a marker of epithelial cells. Bcl‐2 adenovirus E1B‐interacting protein 3 (Bnip3), carbonic anhydrase 9 (CA9), Snail, and transforming growth factor (TGF)‐β1, which are regulated by HIF‐1α, were higher in the aged kidney (Bruick, 2000; Laemmle et al., 2012; Raval et al., 2005; Sowter, Raval, Moore, Ratcliffe, & Harris, 2003; Wykoff et al., 2000; Xu et al., 2015; Zhou et al., 2009), suggesting that the activity of HIF‐1α was increased compared to the young kidney (Figure 1d).

### 2.3 Sirt1 inhibition increased ECM production and apoptosis in HK2 cells

**Figure 2:**
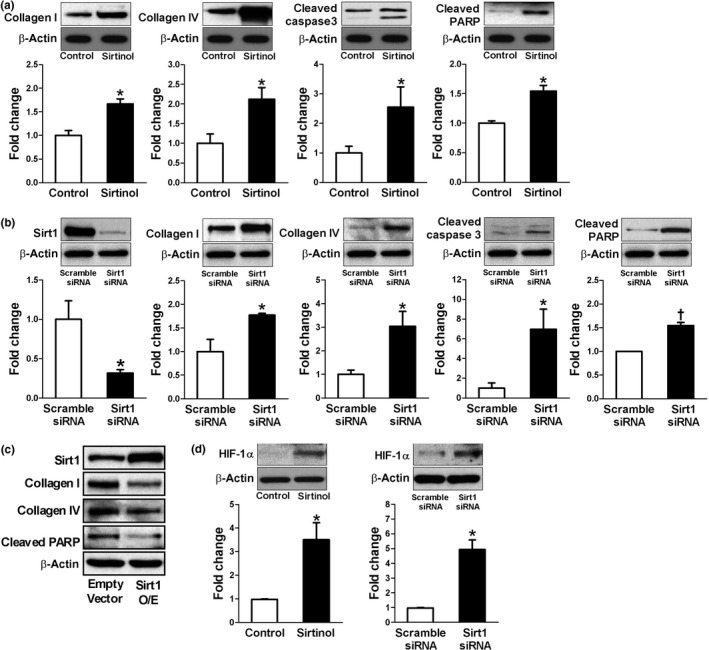
Sirt1 inhibition increases extracellular matrix production and apoptosis in HK2 cells (a, b, and d). Sirt1 was inhibited by sirtinol treatment (10 µM) for 6 hr (*n* = 3) or siRNA (*n* = 3). Representative western blots show the protein levels of collagen I and IV, cleaved caspase‐3, cleaved poly (ADP‐ribose) polymerase (PARP), and HIF‐1α. Effect of Sirt1 overexpression (O/E) was shown in c. *p < 0.05 and ^†^ *p* < 0.001 vs. control

To investigate whether Sirt1 interacts with HIF‐1α in renal tubular epithelial cells, we first examined the effect of Sirt1 expression on extracellular matrix (ECM) production and apoptosis in HK2 cells. As shown in Figure 2a, a Sirt1 inhibitor, sirtinol, significantly induced collagen I and IV production as well as apoptotic markers such as cleaved caspase‐3 and cleaved poly (ADP‐ribose) polymerase (PARP). Similar findings were also observed by siRNA‐mediated knockdown of Sirt1 (Figure 2b). The role of Sirt1 in these changes was further confirmed by the observation that overexpression of Sirt1 induced the opposite changes (Figure 2c). Since we were interested in the interaction of Sirt1 and HIF‐1α, we also tested whether modifying Sirt1 could change HIF‐1α expression even under normoxia. Interestingly, sirtinol or siRNA‐mediated knockdown of Sirt1 significantly enhanced HIF‐1α expression in HK2 cells (Figure 2d).

### 2.4 **Hypoxia decreased Sirt1 expression leading to the acetylation and activation of HIF‐1**α** in HK2 cells**

**Figure 3:**
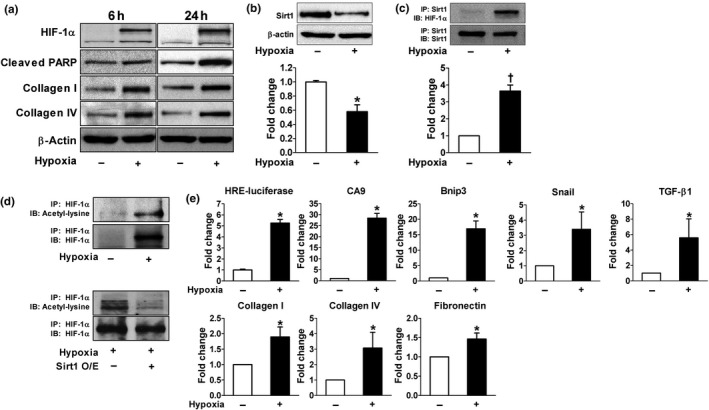
Hypoxia decreases Sirt1 expression leading to the acetylation and activation of HIF‐1α in HK2 cells. Cells were incubated under hypoxia (1% O_2_) for 6 hr (a) or 24 hr (a–e). Representative western blots show the protein levels of HIF‐1α, collagen I and IV, cleaved poly (ADP‐ribose) polymerase (PARP), and Sirt1. Equal amounts of protein were subjected to immunoprecipitation with Sirt1 antibody (c) or HIF‐1α antibody (d) followed by immunoblotting with antibody against Sirt1, HIF‐1α, or acetyl‐lysine, *n* = 6–7. (e) Cells were transfected with luciferase reporter vector for hypoxia‐responsive element (HRE), and luciferase activity was measured, *n* = 3. Real‐time RT–PCR was performed to measure the level of mRNA expressions of carbonic anhydrase 9 (CA9), Bcl‐2 adenovirus E1B‐interacting protein 3 (Bnip3), Snail, TGF‐β1, collagen I and IV, and fibronectin. *n* = 3. *p < 0.001 and ^†^ *p* < 0.01 vs. control

We then analyzed the effect of hypoxia on Sirt1 expression and its interaction with HIF‐1α. During hypoxia, the expression of Sirt1 was significantly reduced whereas HIF‐1α, collagen I/IV, and cleaved PARP were upregulated (Figure 3a,b). The interaction between Sirt1 and HIF‐1α was determined by immunoprecipitation. After endogenous HIF‐1α was induced by hypoxia, Sirt1–HIF‐1α binding was observed (Figure 3c). We next examined whether Sirt1 deacetylates HIF‐1α. Lysyl acetylation of HIF‐1α was detected by immunoblotting with anti‐acetyl‐lysine in HIF‐1α immunoprecipitates. As shown in Figure 3d, Sirt1 overexpression significantly decreased HIF‐1α acetylation suggesting that Sirt1 regulates lysyl acetylation of HIF‐1α. These results suggest that the down‐regulation of Sirt1 induced by hypoxia might lead to increased acetylation of HIF‐1α. To test the functional consequences of HIF‐1α acetylation, HIF‐1α transcriptional activity was measured by luciferase reporter assay and mRNA transcription of HIF‐1 target genes such as CA9, Bnip3, Snail, and TGF‐β1 as well as ECM‐related genes. As shown in Figure 3e, hypoxia‐induced lysyl acetylation of HIF‐1α was associated with markedly increased transcriptional activity of HIF‐1α.

### 2.5 **Sirt1 activation induced deacetylation and inactivation of HIF‐1**α** and subsequently rescued hypoxia‐induced tubular changes**

**Figure 4:**
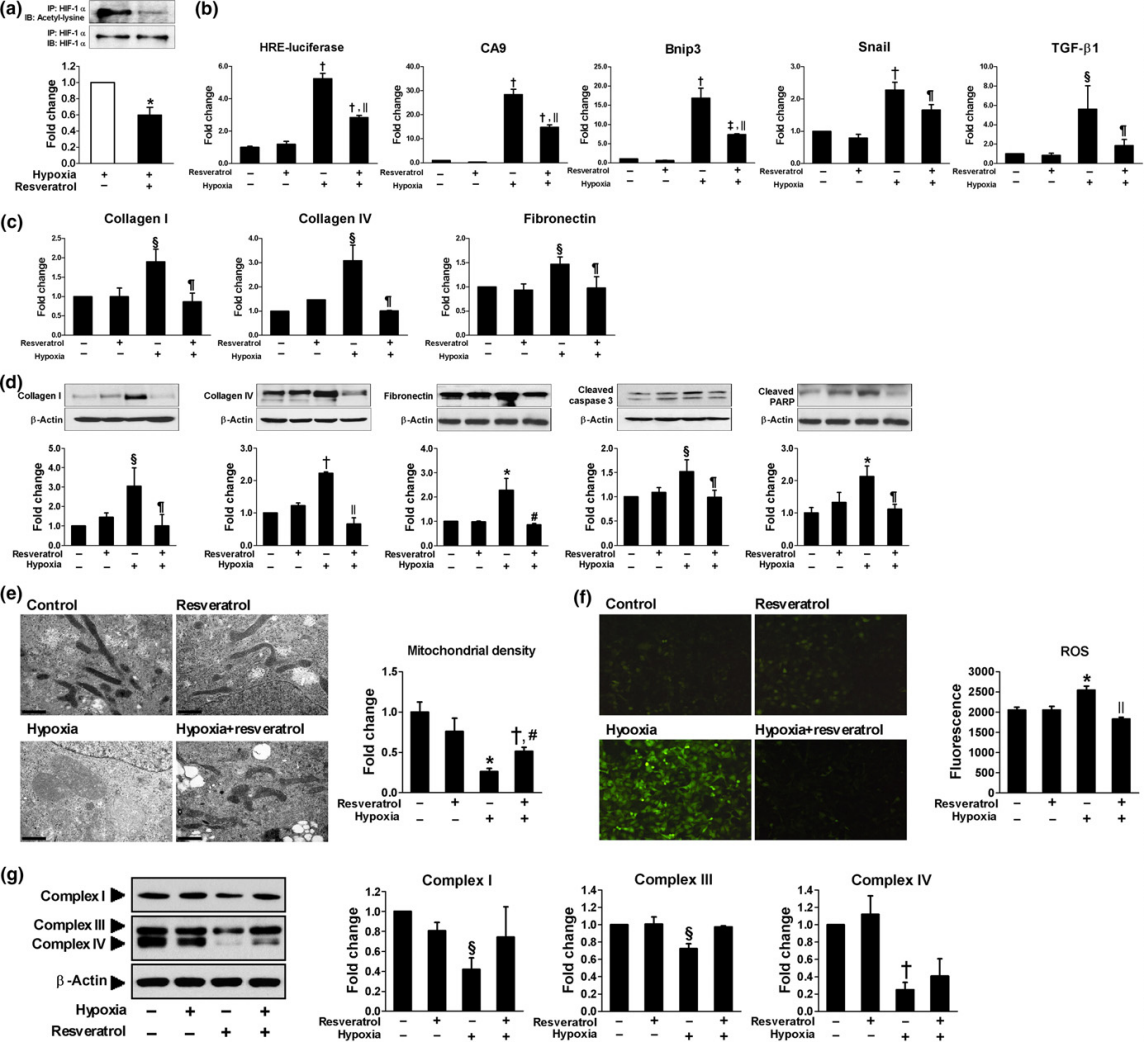
Sirt1 activation induces deacetylation and inactivation of HIF‐1α and subsequently rescues hypoxia‐induced tubular damage. (a–g) Cells were incubated under hypoxia (1% O_2_) for 24 hr in the presence or absence of 10 µM resveratrol pretreatment for 30 min (*n* = 4–8). (a) Equal amounts of protein were subjected to immunoprecipitation with HIF‐1α antibody followed by immunoblotting with antibody against HIF‐1α or acetyl‐lysine. (b, c) Cells were transfected with luciferase reporter vector for hypoxia‐responsive element (HRE), and luciferase activity was measured. Real‐time RT–PCR was performed to measure the level of mRNA expressions of carbonic anhydrase 9 (CA9), Bcl‐2 adenovirus E1B‐interacting protein 3 (Bnip3), Snail, TGF‐β1, collagen I and IV, and fibronectin. (d) Representative western blots show the protein levels of collagen I and IV, fibronectin, cleaved caspase‐3, and cleaved poly (ADP‐ribose) polymerase (PARP). (e) Transmission electron microscopy of representative HK2 cells; bars = 1 µm. (f) Intracellular reactive oxygen species (ROS) production was measured using the oxidation of 2′,7′‐dichlorofluorescein (DCFH). (g) Representative western blots show the protein levels of mitochondrial respiratory chain‐associated proteins. *p < 0.005, ^†^ *p* < 0.001, ^‡^ *p* < 0.01, and ^§^ *p* < 0.05 vs. corresponding control, ^ǁ^ *p* < 0.001, ^¶^ *p* < 0.05, and ^#^ *p* < 0.01 vs. hypoxia

**Figure 5:**
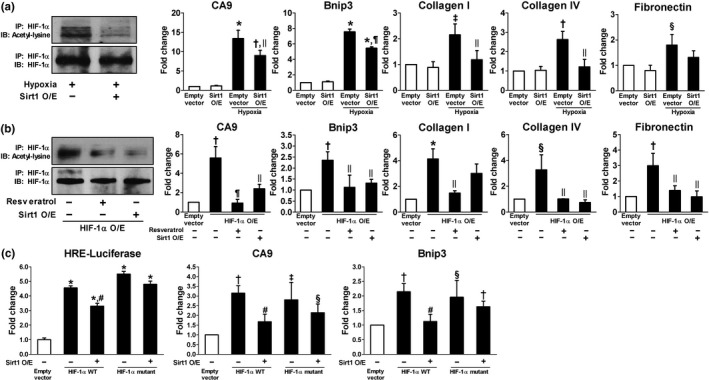
Sirt1 overexpression inhibits HIF‐1α activity. (a) Cells were transfected with Sirt1 or empty vector and subjected to 24 hr hypoxia (1% O_2_), *n* = 3. (b) Cells were transfected with HIF‐1α or empty vector and were pretreated with resveratrol (10 µM) for 30 min or cotransfected with Sirt1, *n* = 6. (c) WT HIF‐1α or K709R HIF‐1α mutant was coexpressed with Sirt1, *n* = 6. Cells were transfected with luciferase reporter vector for hypoxia‐responsive element (HRE), and luciferase activity was measured. (a, b) Equal amounts of protein were subjected to immunoprecipitation with HIF‐1α antibody followed by immunoblotting with antibody against HIF‐1α or acetyl‐lysine. (a–c) Real‐time RT–PCR was performed to measure the level of mRNA expressions of carbonic anhydrase 9 **(**CA9), Bcl‐2 adenovirus E1B‐interacting protein 3 (Bnip3), collagen I and IV, and fibronectin. *p < 0.001, ^†^ *p* < 0.005, ^‡^ *p* < 0.01, and ^§^ *p* < 0.05 vs. empty vector, ^ǁ^ *p* < 0.05, ^¶^ *p* < 0.001, and ^#^ *p* < 0.005 vs. hypoxia or HIF‐1αWT. O/E, overexpression; WT, wild‐type

To examine whether Sirt1 activation deacetylates HIF‐1α and decreases its activity, HK‐2 cells were exposed to hypoxia with or without resveratrol treatment. A Sirt1 activator, resveratrol, decreased acetylation of HIF‐1α which was induced under hypoxia (Figure 4a) and repressed its transcriptional activity assessed by luciferase reporter assay and mRNA transcription of HIF‐1 target genes (Figure 4b). Moreover, hypoxia‐induced production of ECM mRNAs and proteins and apoptosis were significantly restored by resveratrol treatment (Figure 4c,d). Since mitochondrial dysfunction and oxidative stress are well‐known features of ischemic kidney injury, we evaluated mitochondrial morphology and the expression of mitochondrial respiratory chain‐associated proteins. As shown in Figure 4e–g, swollen mitochondria, decreased mitochondrial density, reduced expression of mitochondrial respiratory chain‐associated proteins, and increased reactive oxygen species (ROS) generation were observed in hypoxic HK‐2 cells; however, resveratrol attenuated all of these changes. The inhibition of HIF‐1α activity by Sirt1‐induced deacetylation of HIF‐1α was also confirmed by Sirt1 overexpression under hypoxic condition (Figure 5a) and by resveratrol treatment or Sirt1 overexpression in HIF‐1α ‐transfected HK‐2 cells (Figure 5b). To identify the site of interaction between HIF‐1α and Sirt1, we generated a HIF‐1α vector in which a lysine residue was mutated to an arginine residue (K709R). As shown in Figure 5c, both wild‐type and mutant HIF‐1α significantly enhanced HIF‐1α luciferase activity and mRNA transcription of CA9 and Bnip3. However, the inhibitory effect of Sirt1 overexpression on HIF‐1α activity was only observed in wild‐type HIF‐1α‐transfected cells, but not in mutant HIF‐1α‐transfected cells. These findings indicate that Sirt1 interacts with HIF‐1α, resulting in the deacetylation of HIF‐1α at Lys709.

### 2.6 **Renal tubular cell‐specific HIF‐1**α** overexpression accelerated renal damage**

**Figure 6:**
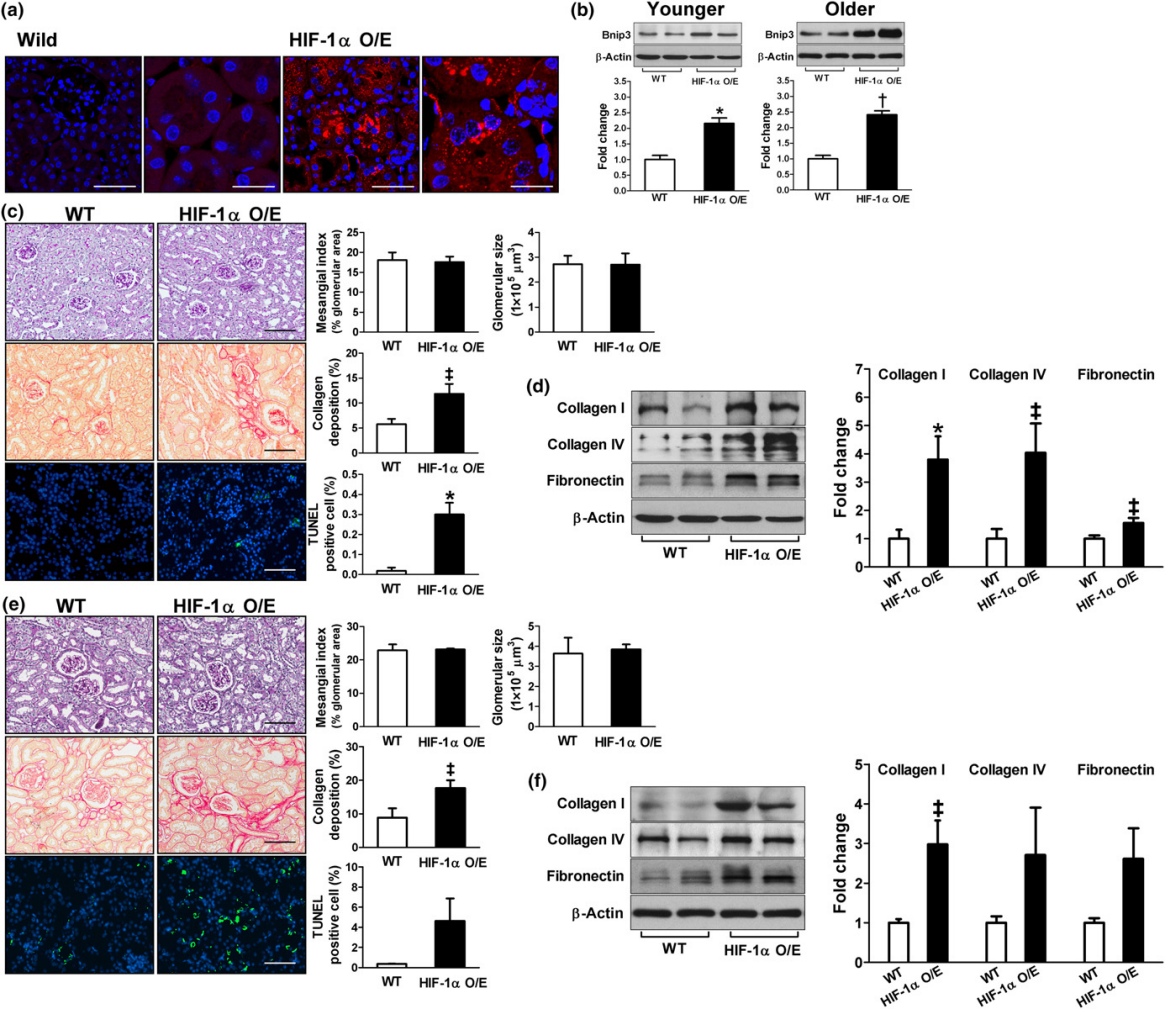
Renal tubular cell‐specific HIF‐1α overexpression (O/E) accelerates renal damage. Six‐week‐old (c, d) or 12‐month‐old (e, f) paired box 8‐reverse tetracycline‐responsive transactivator‐based conditional HIF‐1α transgenic mice and their littermates were treated with doxycycline for 3 days and sacrificed 8 weeks after HIF‐1α induction. (a) Immunofluorescent staining using anti‐HA antibody; bars = 50 and 20 µm, respectively. (b, d, f) Bcl‐2 adenovirus E1B‐interacting protein 3** (**Bnip3), collagen I and IV, and fibronectin protein expression levels were analyzed using immunoblot analysis, *n* = 4–6. (c, e) Representative kidney sections stained with PAS, PicroSirius Red, and TUNEL staining; bars = 100 µm for PAS and PicroSirius Red, 50 µm for TUNEL staining, *n* = 4–7. *p < 0.005, ^†^ *p* < 0.001, and ^‡^ *p* < 0.05 vs. wild‐type (WT)

To assess the role of chronic activation of HIF‐1α on renal damage, we used paired box 8 (Pax8)‐reverse tetracycline‐responsive transactivator (rtTA)‐based conditional HIF‐1α transgenic mice. Six‐week‐old (younger) or 12‐month‐old (older) transgenic mice and their littermates were treated with doxycycline for 3 days and sacrificed 8 weeks after HIF‐1α induction**.** The induction of HIF‐1α‐hemagglutinin (HA) protein expression exclusively in renal tubular epithelial cells was confirmed as shown in Figure 6a. The expression of Bnip3 was significantly higher in both the younger and the older transgenic mice compared to the wild‐type (Figure 6b), suggesting that the activity of HIF‐1α was enhanced in HIF‐1α transgenic mice. Histological analysis exhibited no glomerular changes, but collagen deposition and the number of TUNEL‐positive apoptotic cells in the tubulointerstitial area were significantly higher in the transgenic mice than the wild‐type in both the younger (Figure 6c) and the older groups (Figure 6e). Western blot analyses also confirmed higher expressions of type I/IV collagen and fibronectin in the renal cortex of the transgenic mice compared to the controls (Figure 6d,f). Serum creatinine (0.13 ± 0.01 vs. 0.12 ± 0.01 mg/dl in the younger group; 0.11 ± 0.02 vs. 0.10 ± 0.01 mg/dl in the older group) or cystatin C (0.68 ± 0.02 vs. 0.45 ± 0.02 mg/dl in the younger group; 0.86 ± 0.09 vs. 0.64 ± 0.08 mg/dl in the older group) was not different between the transgenic and the wild‐type mice.

## 3 DISCUSSION

In the current study, we have uncovered that acetylation of HIF‐1α increases its activity and exerts proapoptotic and profibrotic effects in the aged kidney model as well as in hypoxia‐induced tubular responses in vitro. Furthermore, we demonstrated that chronic activation of HIF‐1α specifically in renal tubular epithelial cells promoted apoptosis and fibrosis. Our findings that Sirt1 directly interacts with HIF‐1α, resulting in the deacetylation of HIF‐1α establish a critical regulation of HIF‐1α by Sirt1. We suggest that Sirt1 represses HIF‐1α activity by deacetylation at Lys709. However, under hypoxic stress associated with decreased nicotinamide adenine dinucleotide (NAD^+^) level and NAD^+^/NADH ratio (Braidy et al., 2011; Imai, 2011; Lim et al., 2010), the transcriptional activity of HIF‐1α would be further increased with the induction of acetylation by decreased Sirt1 activity (Nakahata et al., 2008). Therefore, the deficiency of Sirt1 expression or its activity with the upregulation of HIF‐1α observed in the aged kidney could be a key mediator of kidney injury.

Compelling evidence now supports the role of Sirt1 in kidney health and disease. The renal protective effects of Sirt1 have been shown in multiple disease models including aged kidney (Kitada, Kume, Takeda‐Watanabe, Kanasaki, & Koya, 2013; Kume et al., 2010), acute kidney injury induced by ischemia reperfusion **(**Funk & Schnellmann, 2013) or cisplatin (Hasegawa et al., 2010), unilateral ureteral obstruction (UUO; He et al., 2010), and diabetic nephropathy (Chuang et al., 2011; Liu et al., 2014; Shang et al., 2013). However, beneficial effect of Sirt1 activation has not been reported in association with its deacetylation targeting HIF‐1α in the kidney disease. To the best of our knowledge, this is the first study suggesting the role of Sirt1–HIF‐1α interaction in renal tubular epithelial cells particularly in association with renal fibrosis and apoptosis related to aging. The findings of our in vitro study demonstrating that Sirt1 has anti‐apoptotic, anti‐fibrotic, and anti‐oxidative effects were consistent with previous data. These protective effects of Sirt1 were further confirmed by the findings that activation of Sirt1 using resveratrol significantly attenuated hypoxia‐induced apoptosis, ECM production, mitochondrial damage, and ROS generation. A decreased expression of Sirt1 in the aged kidney compared to the control was in accordance with previous studies (Kume et al., 2010; Lim et al., 2012).

A few studies have implicated the link between Sirt1 and HIF proteins, but the resulting outcome of their interaction is still controversial. Dioum et al. (2009) reported that Sirt1 does not target HIF‐1α, rather it targets HIF‐2α in Hep3B or HEK293 cells during hypoxia and that their interaction stimulates the transcriptional activity of HIF‐α2. In contrast, another recent data showed that Sirt1 interacts with HIF‐1α and the deacetylation of HIF‐1α stabilizes HIF‐1α protein expression and promotes its activity using human hepatoma cell lines such as Hep3B, HepG2, and Huh7 cells **(**Laemmle et al., 2012). Unlike these observations, we demonstrated that the interaction between Sirt1 and HIF‐1α inhibits the latter's transcriptional activity, using luciferase reporter assay and mRNA transcription of HIF‐1 target genes in HK2 cells, which is consistent with the results of Lim et al. (2010) shown in HT1080 and HEK293 cells. Interestingly, we found that Sirt1 inhibition using sirtinol or siRNA significantly induced HIF‐1α even without hypoxia in HK2 cells suggesting the involvement of HIF‐1α in the regulation of apoptosis and ECM production under normoxia as well as hypoxia. Induction of HIF‐1α without hypoxic stimuli has also been observed by cytokines and growth factors such as insulin, insulin‐like growth factor, TGF, epidermal growth factor, and platelet‐derived growth factor (Kietzmann, Mennerich, & Dimova, 2016; Pugh & Ratcliffe, 2003). Therefore, it appears that the effect of interaction between Sirt1 and HIF‐1α on the regulation of HIF‐1α activity or its expression is cell type‐specific and remains unclear.

HIF‐1α is a well‐known transcription factor that facilitates adaptive cellular responses to hypoxia. Although HIF‐1α is known to play a key role in the protection from acute ischemia (Bernhardt et al., 2006; Ma et al., 2009; Matsumoto et al., 2003), recent evidence suggested that prolonged unregulated activation of HIF‐1α could enhance maladaptive responses, which lead to glomerulosclerosis and interstitial fibrosis in multiple animal models. Genetic ablation of HIF‐1α in the renal tubular epithelium has been shown to inhibit tubulointerstitial fibrosis in UUO kidneys (Higgins et al., 2007). On the other hand, the stable expression of HIF‐1α by von Hippel–Lindau deletion promoted interstitial fibrosis (Kimura et al., 2008). In a podocyte ablation model, HIF‐1α has been shown to induce glomerulosclerosis through interaction with Smad3 (Baumann, Hayashida, Liang, & Schnaper, 2016). Finally, a recent report has shown that HIF‐1α blockade prevented the development of diabetic nephropathy in type 1 diabetic mice (Nayak et al., 2016). These data suggested HIF‐1α as a mediator of renal damage. Our observations showing that 8‐week activation of HIF‐1α, specifically in renal tubular epithelial cells, promoted apoptosis and ECM accumulation strongly support a deleterious effect of HIF‐1α in renal tubulointerstitial injury.

Considering that hypoxia is the major mechanism involved in the pathogenesis of renal senescence (Nangaku, Inagi, Miyata, & Fujita, 2008), it is expected that HIF‐1α expression or activity would be increased in the aged kidney. Indeed, using pimonidazole immunostaining and hypoxia‐responsive reporter of transgenic rats, Tanaka et al. have identified significant hypoxia in all areas of the aged kidney, especially in the cortical area (Tanaka et al., 2006). They showed a significant correlation between the level of hypoxia and the degree of age‐related tubulointerstitial injury. In this study, we also found a higher expression of HIF‐1α and its downstream targets such as Bnip3, Snail, and TGF‐β1 in the aged kidney cortex. Since Bnip3 is a well‐known player of hypoxia‐induced apoptosis and Snail and TGF‐β1 are the key regulators of epithelial to mesenchymal transition and renal fibrosis, the activation of HIF‐1α‐regulated pathways could mediate renal damage. The significance of our study is highlighted by the findings that acetylated HIF‐1α is increased in the aged kidney and that activation of HIF‐1α induced by acetylation is associated with the development of tubular apoptosis and interstitial fibrosis. Our observations showing that acetylated HIF‐1α was localized in the damaged tubular cells with fragmented nuclei and weak expression of E‐cadherin indicate the correlation of Sirt1–HIF‐1α interaction to damaged area of the aged kidney.

A limitation of our study is that we did not perform in vivo studies using HIF‐1α overexpressed mice to test whether increased Sirt1 activity could rescue the tubulointerstitial damage. Further experiments will need to address this issue.

In conclusion, we have identified a role of Sirt1–HIF‐1α interaction as a key mediator of the aged kidney. Our study suggests that deacetylation of HIF‐1α induced by Sirt1 activation may have a therapeutic benefit to slow kidney damage in the aging process. Further studies will be needed to determine whether acetylated HIF‐1α is also increased in other disease models of kidney injury.

## 4 MATERIALS AND METHODS

### 4.1 Animals

Male C57Bl/6J mice were obtained from the Korea Research Institute of Bioscience and Biotechnology (Ochang, Korea). The mice were housed in a pathogen‐free facility set on a 12‐hr light–dark cycle and given free access to water and regular laboratory chow (Cargill Agri Purina, Gunsan, Korea). We used a transgenic mouse line containing HA‐tagged HIF1dPA, which escapes from recognition by the von Hippel–Lindau tumor suppressor gene product owing to a proline to alanine substitution, preceded by a floxed stop codon cassette, inducing HIF‐1α expression Cre‐dependent (Kim et al., 2006). Mice carrying the HIF1dPA‐HA transgene were bred with mice with the Pax8‐rtTA driver to allow overexpression of HIF‐1α in renal tubular cells (Traykova‐Brauch et al., 2008). Transgenic mice, which carried the three transgenes, were generated by multiple breeding strategies, and progeny without all three transgenes was regarded wild‐type. Given the large number of transgenes and complex breeding strategies, the genetic background strain for all mice used in this study was mixed. HIF‐1α protein expression could be determined by immunofluorescent staining with an anti‐HA antibody (Cell Signaling, Danvers, MA, USA). To obtain renal tubular cell‐specific HIF‐1α‐overexpression at the chosen time of collection, doxycycline (Sigma Chemical Co., St. Louis, MO, USA) was added to the drinking water at a concentration of 2 mg/ml for at least 3 days. Pax8‐rtTA, tetO‐Cre, and HIF1dPA‐HA transgenic mice were obtained from the Samuel Lunenfeld Research Institute at Mount Sinai Hospital, Toronto, ON, Canada. All animal studies were conducted with the approval of the Institutional Care and Use Committee of the Soon Chun Hyang University Hospital and School of Medicine, Ewha Womans University. Our study complied with the National Institutes of Health Guidelines for the Care and Use of Experimental Animals.

### 4.2 Blood and urine chemistry

Serum and urine creatinine, BUN, and serum cystatin C levels were measured using a colorimetric method with a Cobas 8000 analyzer (Roche, Mannheim, Germany). Urinary albumin level was measured using enzyme‐linked immunosorbent assay kit (Abcam, Cambridge, MA, USA). GFR was calculated using the equation (urine creatinine in mg/dl × urine flow rate in dl/hr)/serum creatinine in milligrams per deciliter.

### 4.3 Cell culture

HK2 cells purchased from American Type Culture Collection (Manassas, VA, USA) were cultured at 37°C in a 5% carbon dioxide (CO_2_) atmosphere in DMEM (Life Technologies, Carlsbad, NY, USA) mixed 1:1 (vol:vol) with F12 medium (Life Technologies) supplemented with 10% FBS (Life Technologies). Near confluent cells were incubated with serum‐free media for 24 hr to arrest and synchronize cell cycle. For hypoxic stimuli, cells were incubated in a hypoxic chamber using BD GAS‐PAK EZ anaerobic pouch (BD, Sparks, MD, USA) with 5% CO_2_/1% O_2_ and 94% N_2_ (v/v). For some experiments, cells were pretreated or treated with resveratrol (10 µM; Sigma‐Aldrich, St Louis, MO, USA) or sirtinol (10 µM; Sigma‐Aldrich) for the specified duration.

### 4.4 Transfection and luciferase assay

For transient transfection, subconfluent cells were transfected with 2 μg plasmid of pECE‐flag‐Sirt1 (Addgene, Cambridge, MA, USA), pECE empty vector (Addgene), HA‐HIF‐1α‐wt‐pcDNA3, HA‐HIF‐1‐K709R‐pcDNA3, pGL2‐hypoxia‐responsive element (HRE) plasmid (Addgene) using Trans IT 2020 (Mirus Bio LLC, Madison, WI) following the manufacturer's instructions. For luciferase assay, pGL2 luciferase reporter vector (Addgene) for HRE was cotransfected with Firefly and Gaussia luciferase control plasmid using a Dual Luciferase Assay kit (Thermo Scientific, Rockford, IL, USA). Luciferase activity was measured using a luminometer.

### 4.5 siRNA

An effective predesigned siRNA of Sirt1 (Applied Biosystems, Carlsbad, CA, USA) was selected in a preliminary study. The sequence was as follows: sense 5ʹ‐CCCUCAAAGUAAGACCAGUTT‐3ʹ and antisense 5ʹ‐ACUGGUCUUACUUUGAGGGAA‐3ʹ. Cells were transfected with siRNA (50 nM/well) using Lipofectamine RNAiMAX (Life Technologies) reagent under serum‐ and antibiotic‐free condition for 24 hr.

### 4.6 RT‐PCR

Total RNA was prepared using TRIzol reagent (Life Technologies). The cDNA product was synthesized and amplified by PCR using a StepOne Plus Real‐Time PCR System (Applied Biosystems). For PCR, we used TaqMan Assays‐on‐Demand Gene Expression Products from Applied Biosystems: Bnip3, Hs00969291_m1; CA9, Hs00154208_m1; Snail, Hs00195591_m1; TGF‐β1, Hs00998133_m1; collagen I, Hs00164004_m1; collagen IV, Hs01022505_m1; and fibronectin, Hs01549976_m1. As an internal control, 18S ribosomal RNA expression, Hs99999901_s1, was quantified with the target genes.

### 4.7 Western blot analysis

Tissue and cell lysates were subjected to western blot analysis using a standard procedure. Membranes were immunoblotted with antibodies against collagen I/IV (Southern Biotech, Birmingham, AL), fibronectin (Abcam, Cambridge, UK), Sirt1 (Cell Signaling), Bnip3 (Abcam), CA9 (Abcam), TGF‐β1 (Abcam), HIF‐1α (Cell Signaling), cleaved caspase‐3 (Cell signaling), cleaved PARP (Cell Signaling), mitochondrial complexes I, III, and IV (Abcam), and β‐actin (Sigma‐Aldrich), followed by appropriate secondary antibodies.

### 4.8 Immunoprecipitation

Approximately 1 mg of total proteins was incubated overnight at 4°C with anti‐Sirt1 antibody (Cell Signaling) or anti‐HIF‐1α antibody (Novus Biologicals, Littleton, CO, USA) followed by precipitation with 20 µl of protein A/G‐Plus‐Agarose (Santa Cruz, Dallas, TX, USA) for 4 hr at 4°C. The precipitated complexes were immunoblotted with anti‐Sirt1 (Cell Signaling), anti‐HIF‐1α (Novus Biologicals), or anti‐acetyl‐lysine (Cell Signaling).

### 4.9 Histology

Paraffin‐embedded sections (3 µm) were subjected to periodic acid–Schiff (PAS), Masson's trichrome (Sigma‐Aldrich), and PicroSirius Red staining (Abcam). On PAS‐stained kidney sections, 150 different superficial glomeruli were randomly sampled for morphometric analysis and the percentage mesangial area was determined using the Image Scope software (Aperio, Vista, CA). Collagen deposition was determined on the trichrome‐stained or PicroSirius Red‐stained kidney sections. The positive area was quantitatively measured using the Image Scope software. For the analysis of macrophage infiltration, paraffin‐embedded sections were stained for CD68 (Abcam). Diaminobenzidine was used for visualization of immunoreactivity followed by hematoxylin for nuclear counterstaining.

Immunofluorescence staining was performed using paraffin‐embedded sections (3 µm). After blocking with 10% normal goat serum for 1 hr, the sections were incubated overnight at 4℃ with anti‐HA (Cell Signaling), anti‐HIF‐1α (Novus Biologicals), anti‐acetyl‐lysine (Cell Signaling), and anti‐E‐cadherin (BD) antibodies. Antibody staining was visualized with Alexa Fluor 488 goat anti‐mouse, Alexa 568 goat anti‐rabbit, or Alexa 647 goat anti‐mouse. Counterstaining of cell nuclei was carried out with DAPI (Sigma‐Aldrich) for 10 min. Slides were viewed with a confocal microscope (LSM 700; Carl Zeiss, Oberkochen, Germany). Detection of apoptotic cells in paraffin‐embedded kidney sections was performed using In situ Cell Death Detection kit (Roche, Indianapolis, IN, USA) according to the manufacturer's instructions.

### 4.10 Transmission electron microscopy

HK‐2 cells were postfixed with 2.5% glutaraldehyde and 1% OsO_4_ (in 0.1 M phosphate buffer, pH 7.4), dehydrated, and embedded in Poly/Bed 812 resin. Ultrathin sections were photographed using a JEM‐1010 transmission electron microscope (JEOL, Tokyo, Japan). Mitochondrial density was determined by measuring the average matrix density value relative to the surrounding cytoplasm (Chaudhuri, Artiga, Abiria, & Clapham, 2016). Since damaged mitochondrial matrices lost their contents, the density was closer to or lighter than cytoplasm as compared to normal mitochondria.

### 4.11 Intracellular ROS

Intracellular ROS production was measured as previously described (Ha, Yu, Choi, Kitamura, & Lee, 2002). HK‐2 cells were washed with Dulbecco's phosphate‐buffered saline and incubated in the dark for 5 min in Krebs–Ringer solution containing 5 µM 5‐(and‐6)‐chloromethyl‐2′,7′‐dichlorodihydrofluorescein diacetate (CM‐H2DCFDA; Molecular Probes Inc., Eugene, OR, USA). ROS generation was detected as a result of the oxidation of 2′,7′‐dichlorofluorescein (DCFH) at 488 nm excitation and 515–540 nm emission under a fluorescence microscope (Leica, Wetzlar, Germany).

### 4.12 Statistical analyses

The mean values were compared using ANOVA followed by Fisher's least significant difference method. Unpaired two‐tailed Student's *t* tests were used where appropriate. Data are presented as the mean ± standard error (*SE*). A *p* value <0.05 was considered statistically significant.

## CONFLICT OF INTEREST

None.

## AUTHOR CONTRIBUTIONS

DRR, MRY, and KHK performed the experimental works. HK, SHK, JSJ, and DCH analyzed the data. HN designed the study, analyzed the data, and wrote the manuscript.
